# Dogs infected by *Dirofilaria immitis*: a threat to the health of human and non-human animals in Rio de Janeiro, Brazil

**DOI:** 10.29374/2527-2179.bjvm001723

**Published:** 2023-07-25

**Authors:** Bruno Alberigi, Esmael Carvalho, Flavya Mendes-de-Almeida, Norma Labarthe, Fabio Barbour Scott

**Affiliations:** 1 Veterinarian, DSc. Departamento de Medicina e Cirurgia Veterinária (DMCV), Instituto de Veterinária (IV), Universidade Federal Rural do Rio de Janeiro (UFRRJ). Seropédica, RJ, Brazil.; 2 Undergraduate in Biomedicine, UNIASSELVI, São Gonçalo, RJ, Brazil.; 3 Veterinarian, DSc. Departamento de Patologia e Clínica Veterinária, Universidade Federal Fluminense, Niterói, RJ, Brazil.; 4 Veterinarian, DSc, Programa de Pós-Graduação em Ética, Bioética e Saúde Coletiva, Fundação Oswaldo Cruz, Rio de Janeiro, RJ, Brazil.; 5 Veterinarian, DSc. Departamento de Parasitologia Animal, IV, UFRRJ, Seropédica, RJ, Brazil.

**Keywords:** heartworm, microfilariae, transmission, one health, dirofilariose, microfilária, transmissão, saúde única

## Abstract

This study aimed to investigate the presence of *Dirofilaria immitis* microfilaremia in dogs from two regions of the state of Rio de Janeiro, Brazil, where heartworm infections are highly prevalent. Blood samples were collected from dogs aged > 12 months, independent of the use of preventatives. All samples obtained and analyzed using Knott's modified test by the investigators. A total of 133 blood samples were tested, and *D. immitis* microfilariae were detected in 29 of them, resulting in an occurrence of 21.8%. The percentage of dogs with microfilaremia detected raises concerns for pet families, one health professionals, and small animal practitioners. Microfilaremic dogs are the richest source of infection for the mosquitoes, increasing the risk of transmission. Therefore, the stakeholders in One Health must raise concerns regarding the health of wild animals, as wild canids and other species of wild animals are exposed to the risk of *D. immitis* infection. In addition, humans can get infected and develop human pulmonary dirofilariasis. In conclusion, the presence of dogs with microfilaremia potentiates opportunities for *D. immitis* transmission, exposing all animals, wild or domestic, human or non-human to the disease.

## Resumo

Este estudo objetivou investigar a presença de cães portadores de microfilemia da espécie *Dirofilaria immitis* em duas áreas do estado do Rio de Janeiro, Brasil onde a infecções por vermes do coração são altamente prevalescentes. Amostras de sangue foram obtidas de cães com mais de 12 meses de idade, independentemente do uso de preventivos. Todas as amostras foram obtidas e analisadas pelo grupo do estudo utilizando-se o teste de Knott modificado. Um total de 133 amostras foi testado e microfilárias de *D. immitis* foram detectadas em 29 amostras, resultando em uma ocorrencia de 21,8%. A porcentagem de cães com microfilaremia detectada gera preocupações para as famílias de animais de estimação, para profissionais da saúde única e para clínicos de pequenos animais. Cães microfilarêmicos são a mais rica fonte de infecção para mosquitos, aumentando o perigo de transmissão. Assim, atores de todos os segmentos sociais com interesse na saúde única devem incluir a saude de animais selvagens em suas preocupações, uma vez que canideos selvagens e outras espécies de animais selvagens são expostos ao desafio da infecção por *D.immitis.* Além disso, seres humanos podem se tornar infectados e desevolver dirofilariose pulmonar humana. Em conclusão, a presença de cães microfilarêmicos potencializa as oportunidades de trasmissão de *D. immitis*, expondo todos os animais, selvagens ou domesticos, humanos ou não humanos à doença.

Mosquito-borne *Dirofilaria immitis* infection causes canine cardiopulmonary disease that is worsened by the endosymbiont bacteria *Wolbachia* sp. (Boreham & Atwell, 1998; Kramer et al., 2005). Adult worms inhabit the pulmonary arteries and right chambers of the heart, where they mate and produce the first-stage larvae called microfilariae. When a mosquito receives a blood meal from a microfilaremic canid, the microfilariae infect the mosquito and undergo two molts. The infective third-stage larvae (L3) migrate to the head of the mosquito, and when the infected mosquito feeds on another animal, they migrate to the skin and actively infect the new host (Lok, 1988). Because canids are the best-adapted hosts for *D. immitis*, they are also the richest source of microfilariae for mosquitoes (Hays et al., 2020).

Updates on infection rates in the state of Rio de Janeiro mostly report the presence of adult *D. immitis* antigens in canine blood samples, while there remains inadequate information on the ability of microfilariae to infect mosquitoes. The latest reports based on antigen detection show that 53.9% of dogs were infected in the focus area of the eastern lowlands of the state (Labarthe et al., 2014). In the western area of metropolitan Rio de Janeiro, the prevalence of antigenemic dogs was 21.6% (Moraes-da-Silva et al., 2016), and when the infection was surveyed by the detection of antigens in different areas of the city of Rio de Janeiro, it was 7% (Mendes-de-Almeida et al., 2021).

The density of infective mosquitoes (carrying L3) tends to increase as the abundance of microfilaremic dogs increase, especially because mosquitoes are more attracted to *D. immitis* infected dogs than non-infected ones (Mckay et al., 2013; Zohdy et al., 2019). Therefore, the presence of microfilaremic dogs living in the same neighborhood as uninfected dogs and cats receiving no preventatives increases the risk of transmission.

Considering the increasing threat posed by microfilaremic infection, the aim of this study was to gather information on the prevalence of *D. immitis* microfilaremia in dogs in the state of Rio de Janeiro, Brazil.

**Figure 1:**
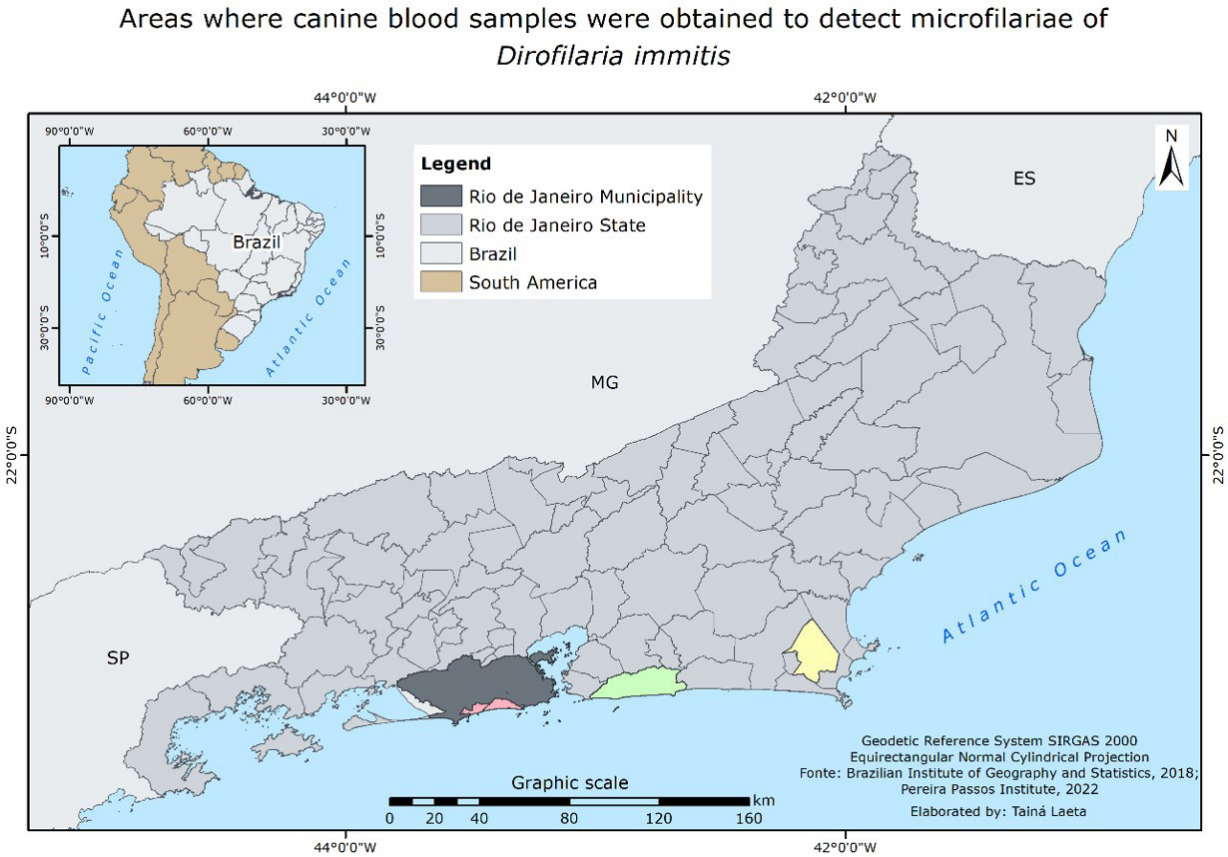
Map of the state of Rio de Janeiro, Brazil showing the areas from where canine blood samples were obtained to detect microfilariae of *Dirofilaria immitis*. The western area of the metropolitan Rio de Janeiro (pink) and the municipalities of Maricá (green) and of São Pedro da Aldeia (yellow), located at the eastern lowland area of the state.

This study evaluated the blood samples collected from dogs living in two different areas of the state of Rio de Janeiro, where the prevalence of heartworm infection is known to be high. These geographical areas included the largest municipality (Rio de Janeiro) and two smaller municipalities located in the eastern lowlands (Maricá and São Pedro da Aldeia) (Figure 1).

Dogs were sampled between June and December 2022 and enrolled after obtaining consent from the dog owners. Owners living in the western area of metropolitan Rio de Janeiro (WMRJ) or in the eastern lowland area of the state of Rio de Janeiro (ELRJ) were invited by their veterinarians to bring their dogs for examination by the investigators of the present study. Blood samples were obtained from dogs aged > 12 months, independent of the use of preventatives, and all samples were analyzed using Knott’s modified test (Newton & Wright, 1956).

In total, 133 blood samples were tested: 80 from the WMRJ area and 53 from the ELRJ area (Maricá and São Pedro da Aldeia). *D. immitis* microfilariae were detected in 6 samples from WMRJ dogs (7.5%) and 23 samples from ELRJ dogs (43.4%). The overall occurrence of microfilaremic canines in the studied regions was 21.8%.

The percentage of dogs with microfilaremia detected in the state (21.8%) exhibits a dilemma that demands strong action from pet families, stakeholders in One Health, and small animal practitioners.

Pet families must take into consideration the threat posed by *D. immitis* infection when deciding whether they want to keep their pets healthy. In this case, they must be aware that avoiding heartworm infection will increase the annual medical costs of the pet and demand attention and compliance with veterinarians’ prescriptions (DiGangi, 2020).

One Health stakeholders must raise concerns regarding the health of wild animals. They must be aware that the use of preventatives in the wild is almost impossible and that infective mosquitoes may fly large distances (Verdonschot & Besse-Lototskaya, 2014). To make matters worse, wild canids tend to forage in anthropic areas where they can interact with mosquito-vectors that have fed on microfilaremic dogs and already harbor L3s (Ionică et al., 2016; Magi et al., 2008; Paras et al., 2012). Once microfilaremic, these wild canids become a source of infection to sylvatic mosquito vectors all over their living area, spreading the parasite in the wild. In addition, humans can become infected and develop human pulmonary dirofilariasis (Simón et al., 2003), indicating that *D. immitis* is a threat to many different animal groups, including humans.

Small animal practitioners, on the other hand, have the responsibility of informing their clients about the risk of infection, how it can compromise the pets’ welfare and how it threatens their health (Ryan et al., 2019). Considering that all dogs included in the present survey were eventually seen by veterinarians, it is possible to infer that preventative products are not recommended by veterinarians in a convincing manner or that pet owners choose to run the risk, hoping that their pets will not get infected. In addition, it may indicate that veterinarians do not believe in *D. immitis* infection risk and consequently do not recommend heartworm preventatives.

The high percentage of microfilaremic dogs in the state calls attention to the urgent need for a wide and strong educating task force on *D. immitis* and the threat it represents for human, non-human, wild, or domestic animals. Overall, this study emphasizes the need for increased awareness and action to prevent the spread of heartworm disease in the state of Rio de Janeiro, Brazil, especially considering the contemporary issues of One Health.

This study reported a high prevalence of *D. immitis* microfilaremia in dogs in the state of Rio de Janeiro, Brazil. These results highlight the importance of preventative measures to reduce heartworm transmission, especially in the wake of the growing One Health concept.
